# Initiating cardiopulmonary bypass using a dry venous line: implications and analysis

**DOI:** 10.1051/ject/2025034

**Published:** 2026-03-13

**Authors:** Tristan Benedict, Robert Brownlee, Christopher Foley, Nathan Hoyer, Laura Dell’Aiera, Mary Dooley, Dave Fitzgerald

**Affiliations:** 1 Medical University of South Carolina, College of Health Professions 179 Ashley Ave. Charleston SC 29425 USA

**Keywords:** Cardiopulmonary bypass, Gaseous microemboli, Dry venous line, VAVD, Vacuum-assisted venous drainage, Initiation

## Abstract

*Background*: Autologous priming of the cardiopulmonary bypass (CPB) circuit is a critical technique for reducing hemodilution during cardiac surgery. Vacuum-assisted venous drainage (VAVD) offers access to an alternative method using a dry venous line, aiming to reduce hemodilution associated with crystalloid priming. *Methods*: This study investigates the impact of initiating CPB with a dry venous line on gaseous microemboli (GME) production, compared to a traditional primed venous line in an adult CPB circuit. Using a controlled experimental setup with an oxygenator featuring an integrated arterial filter, we examined GME counts and sizes throughout the circuit under varying VAVD pressures and initiation techniques. *Results*: Results show that higher VAVD pressures and the immediate initiation of CPB correlate with increased GME production post-oxygenator. Statistical analysis reveals significant differences in GME counts and sizes between control and experimental groups. *Conclusion*: The statistical differences in GME size and count observed between initiation types and pressures emphasize the importance of optimal CPB initiation strategies to minimize GME transmission. These findings underscore the need for further research to refine CPB techniques and enhance patient safety in cardiac surgery.

## Introduction

Since the advent of cardiopulmonary bypass (CPB), venous return to the reservoir upon initiation of the bypass has been accomplished through a gravity siphon with a primed (typically via a crystalloid solution) venous line [1]. Vacuum-assisted venous drainage (VAVD) has made the initiation of bypass with a dry venous line possible. A dry venous line technique is utilized prior to initiation, where crystalloid in the venous tubing is drained into the reservoir, and then shuttled into a bag where it would not reach the patient. The decision to utilize a primed or dry venous line is largely based on surgeon preference, and each method has benefits and drawbacks. One concern with a primed venous line is the increase in hemodilution, which is known to decrease hematocrit and increase the risk of allogeneic red blood cell (RBC) transfusion [2]. Venous tubing with ½ inch Inner Diameter (ID) is most utilized in adult patients, and for every foot of venous tubing, a patient will receive about 40 mL of crystalloid. Reducing prime volume in the circuit as an indication for blood conservation is a Class I, Level B recommendation by the Society of Thoracic Surgeons (STS), Society of Cardiovascular Anesthesiologists (SCA), American Society of Extracorporeal Technology (AmSECT), and Society for the Advancement of Blood Management (SABM) [3]. Dickinson and colleagues performed a study where 20,000 patients had their net prime volumes indexed to their body surface area and found that patients with a greater indexed prime volume (4th quartile) were 2.9 times more likely to receive RBC transfusions compared to patients with lower indexed prime volume (1st quartile) [2].

Utilizing a dry venous line, which requires VAVD, eliminates the hemodilution that results from a primed venous line. However, this technique has drawbacks as well, most notably an increase in gaseous microemboli (GME). Hudacko and colleagues performed a laboratory study to compare the detection of GME in a pediatric circuit utilizing a primed venous line and a dry venous line with varying levels of VAVD pressures (−10, −20, and −40 mmHg) [4]. Their results found that a primed venous line resulted in the least amount of GME production. However, the significant increase in GME post-arterial line filter was only found in the −40 mmHg VAVD group. Microemboli, including GME, delivered to patients are known to have adverse effects such as neurological deficits. Pugsley and colleagues performed a randomized controlled trial on patients undergoing CPB that compared microemboli detection through a transcranial Doppler with and without the use of an arterial line filter [5]. They found that patients with fewer microemboli detected were significantly less likely to have cognitive impairment after surgery. Data on the use of a dry venous technique is not readily available; however, the use of dry venous lines is not 0%.

One way to utilize a primed venous line while also reducing hemodilution is by using venous autologous priming (VAP). With VAP, the crystalloid is shuttled into a bag in a controlled manner by displacing it with the patient’s blood. Utilizing this technique whenever possible is a Class I, Level B recommendation [3]. While this option reduces both hemodilution and GME production, some patients may not be able to tolerate VAP due to becoming hemodynamically compromised from blood loss during the VAP process. In this instance, utilizing a dry venous technique can reduce hemodilution while keeping the patient hemodynamically stable without the need for administering vasopressors.

While the study by Hudacko and colleagues compared GME production in a dry venous line versus a primed venous line, this study was done on a pediatric circuit with an oxygenator lacking an integrated arterial line filter [4]. Our study is the first to look at GME production in a dry versus primed venous line with an adult circuit and associated components. This includes the utilization of a modern oxygenator with an integrated arterial line filter. We hypothesized that there would not be a significant difference in GME (post-oxygenator) with the use of a dry venous line compared to a primed venous line upon initiation of CPB. Additionally, we explored the effects of VAVD pressures and CPB initiation techniques on GME variation. If a dry venous technique yields acceptable levels of GME, its use could be another strategy in a multimodal approach to reduce hemodilution. If there is a significant increase in GME levels, this could potentially influence clinical practice and reduce GME reaching patients.

## Materials and methods

### Circuit components

To execute the trials of this study, several considerations and pieces of equipment were necessary. A hypobaric oxygenator was needed to act as the patient, so GME was not recirculated and counted more than once. To construct this hypobaric oxygenator, a LivaNova (London, UK) Inspire 8F was sealed off by applying a sealant to all entrances of the oxygenator besides the blood inlet port, the blood outlet port, and the gas line inlet (which we used to introduce a negative pressure on the oxygenator to pull air). The hypobaric oxygenator was the only makeshift component needed for these trials.

Additional components consisted of a Terumo (Ann Arbor, MI, USA) System 1 heart-lung machine with an arterial roller head, arterial and venous temperature probes, a vacuum pressure line, an arterial pressure line, and tubing clamps. The circuitry used was the LivaNova (London, UK) reservoir and 8F oxygenator, a 3-port manifold, the hypobaric oxygenator, a Medtronic (Dublin, Ireland) reservoir, a vacuum source, and the Emboli Detection and Classification (EDAC) air quantifier (LUNA Innovations, Blacksburg, VA) with 3 measuring probes.

Pressure lines were used for the pressure transducers, factory shunt lines were used to circulate through the manifold as well as the oxygenator purge, and the main circuitry connecting all of the components was ⅜ inch tubing with luge tubing expanding to ½ inch within the arterial roller head. The hypobaric oxygenator outlet was connected to the venous inlet of the LivaNova reservoir by 6 feet of ½ inch tubing. At the start of the 6 feet of ½ inch venous tubing, a quick-connect attachment was placed to easily drain the venous line for all dry trials. The last component was a small heater-cooler unit connected to the oxygenator. The fluid used in this study was 0.9% normal saline with 1342 mL for the control prime volume and 1102 mL for the dry venous line prime volume.

To count and quantify GME, the EDAC transducer emits ultrasonic pulses every 1 ms that are reflected by emboli in the blood as they pass through the EDAC connector. The amplitude of the reflected signals is then analyzed to determine bubble size, count, and calculated volume (total embolic load).

### Circuit setup

**Figure 1:**
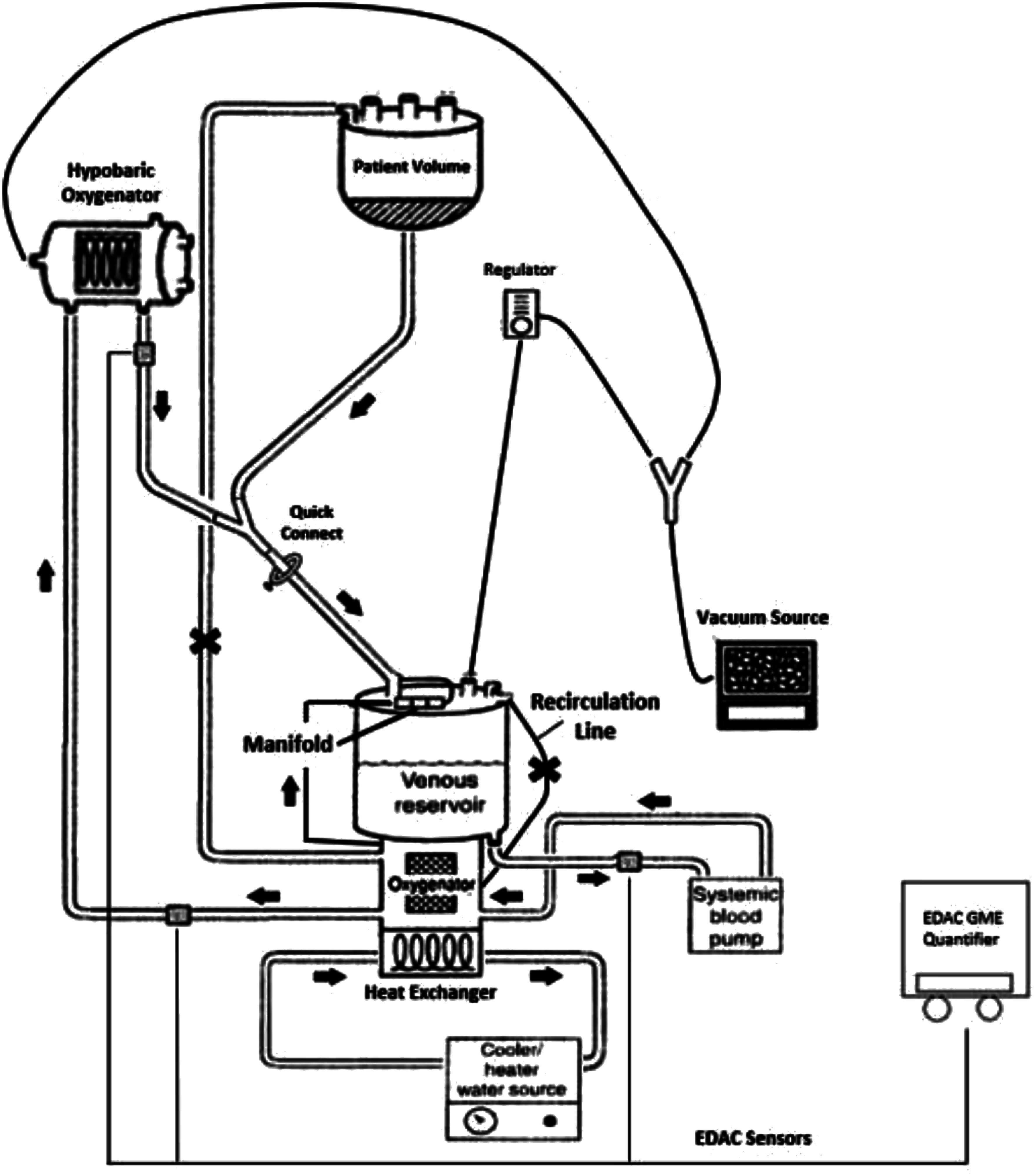
Circuit design.

The circuit setup emulated a standard bypass configuration, commencing with the LivaNova reservoir. Fluid was drawn through the luge tubing in the arterial roller head and propelled through the LivaNova Inspire 8F oxygenator, with the manifold line open and the purge line closed. The fluid was then pumped through 3/8-inch tubing into the hypobaric oxygenator to remove any GME present in the system (Figure 1).

After exiting the hypobaric oxygenator, the fluid was directed through a section of 3/8-inch tubing, leading to a ⅜-⅜-⅜ inch Y connector. The fluid was pushed straight through the Y connector past the quick-connect, through a ⅜-½ connector to a 6-foot length of ½-inch tubing back to the venous inlet of the LivaNova reservoir (Figure 1).

On the other port of the ⅜-inch Y connector, a Medtronic reservoir was mounted at the top of the heart-lung machine to simulate patient volume (1 L) entering the circuit. Temperature probes were positioned at the venous inlet of the LivaNova reservoir and the arterial outlet of the LivaNova 8F oxygenator. The vacuum source was split into two lines, one was connected to the hypobaric oxygenator set at −800 mmHg, while the other line ran to a regulator and into the venous reservoir to maintain controlled negative pressure (Figure 1).

Within the circuit, 3 EDAC sensors were strategically placed. The first was positioned post-LivaNova reservoir and pre-arterial roller head. The second was placed post-LivaNova Inspire 8F oxygenator and pre-hypobaric oxygenator, while the third was positioned post-hypobaric oxygenator (Figure 1).

For volume reset into the Medtronic reservoir, a ⅜-inch line was connected from the cardioplegia port of the oxygenator to the top of the Medtronic reservoir. This line remained clamped during all trials and served solely for resetting volume.

After configuring the circuit, each trial was organized using one of three distinct methods. The control trials featured a fully primed venous line without any negative pressure applied to the reservoir. In the subsequent set of trials, the circuit was primed, then clamped off, and the 6-foot venous line was drained into the venous reservoir. With the venous line now devoid of fluid, the prime volume from the venous line was directed into an empty bag, ensuring that the fluid level in the reservoir matched the starting level of the control trials (300 mL). This procedure was carried out for every trial apart from the control trials. The other two setups with the dry venous lines were distinct by their starting VAVD pressures: −20 mmHg and −40 mmHg in the venous reservoir.

### Procedures

For this study, the Medtronic venous reservoir was mounted above the circuit and Y’d into the 6-foot venous line to simulate patient volume entering the circuit. This will be referred to as the “patient reservoir.” In the study trials, there were two separate initiation techniques. One called “Instant Initiation” where the arterial and venous lines were unclamped and the arterial pump head was turned on before the venous line was filled, keeping the reservoir level between 200 and 300 mL. This technique was also used for control trials. The second technique is called “Delayed Initiation,” where the venous line was unclamped first, the venous line was filled with volume from the “patient” reservoir until the level rose to 400 mL, then the arterial line was unclamped and the arterial pump head was started. The Medtronic “patient reservoir” contained 1 L of volume for all trials. The oxygenator purge remained closed during all trials, and the manifold line was opened during every trial when 1LPM of flow was obtained. All trials were conducted at 37 °C for 3 min with an arterial line pressure between 130 and 150 mmHg. A flow of 5 LPM was acquired within 10 s of initiation, regardless of the initiation technique. EDAC sensors were placed post-reservoir, post-oxygenator, and post-hypobaric oxygenator for every trial for GME measurements. Each trial was reset by clamping the arterial outlet of the oxygenator and forcing volume through the cardioplegia port and into the Medtronic reservoir until volume levels were back to baseline.

### Trial groups

The first 10 trials were the control trials. These trials underwent the “Instant Initiation” technique, with no negative pressure on the venous reservoir and a primed venous line. The second set of 10 trials used a dry venous line with instant initiation and a pressure of −20 mmHg on the venous reservoir (“I(−20)”). The third set of 10 trials had the dry venous line with Instant Initiation and a pressure of −40 mmHg on the venous reservoir (“I(−40)”). The fourth set of 10 trials had the dry venous line with delayed initiation and a pressure of −20 mmHg on the venous reservoir (“D(−20)”). The fifth set of 10 trials had the dry venous line with delayed initiation and a pressure of −40 mmHg on the venous reservoir (“D(−40)”).

### Data analytic strategy

With the EDAC recording during the 3-minute circulation time, GMEs were counted by size and quantity in strategic locations on the circuit. The post-venous reservoir sensor counted and sized GME coming in from the venous line. The sensor post-oxygenator counted and sized GME, leaving the Inspire 8F oxygenator, which is indicative of GME transmission to the patient. The third sensor post-hypobaric oxygenator was necessary for reassurance that GME was exiting the circuit and that no GME was double counted in the trials. The measurements obtained from the EDAC were extrapolated into a data file and compared across a variety of analytical tests.

The results for all trials at each EDAC sensor location include average total GME count and size after 3 min of circulation; however, most GME detection occurred within the first few seconds of the trial. Given the small sample sizes and non-normal distribution of data, differences were evaluated using the nonparametric Kruskal-Wallis test. To assess pairwise differences, the Dwass, Steel, Critchlow-Fligner (DSCF) test was conducted to adjust *P*-values to account for multiple comparisons. The level of significance was assessed at 5%. Data was analyzed using SAS 9.4 (SAS Institute, Cary, NC).

## Results

**Figure 2:**
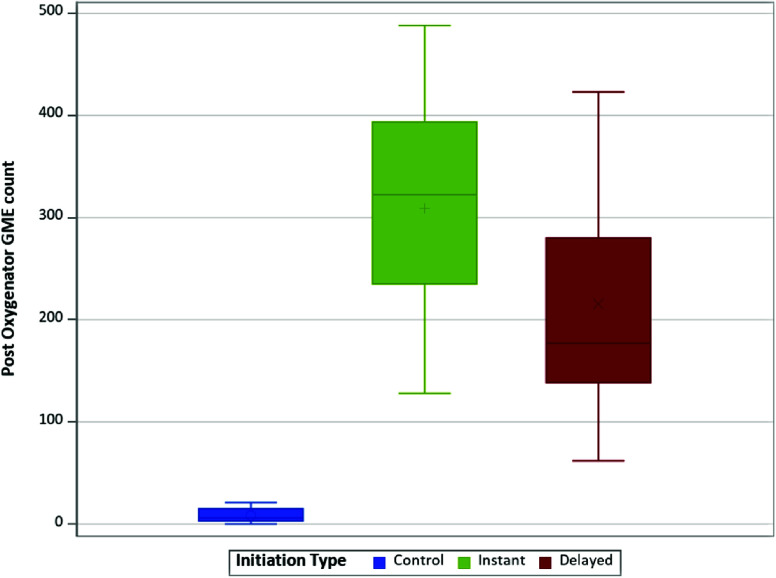
Distribution of post-oxygenator GME count by initiation type.

**Figure 3:**
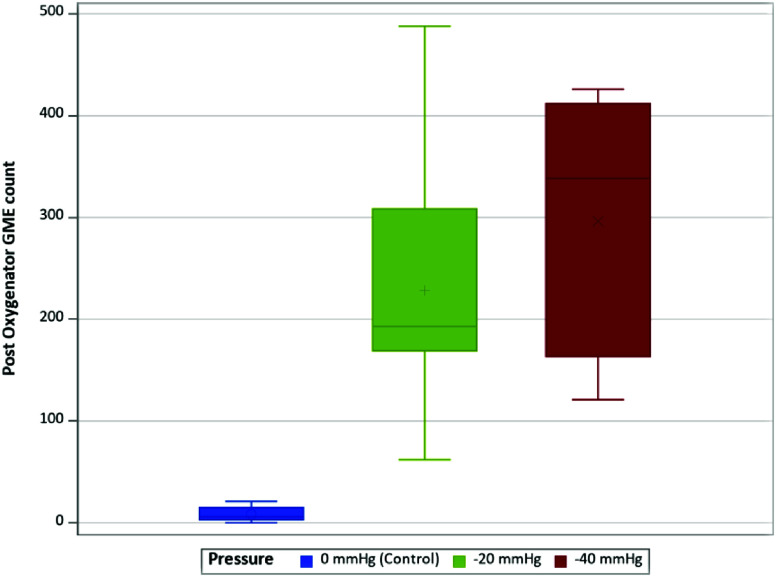
Distribution of post-oxygenator GME count by pressure type.

The distribution of average post-oxygenator GME count varied across different experimental conditions, with a median count of 6.0 for control to 390.5 for I(−40). The highest counts were observed occurring consistently at pressures of −40 mmHg within instant and delayed initiation groups (Table 1). When comparing the average post-oxygenator GME count based on initiation types (Figure 2), there was a statistically significant difference in median count between types (*p* < 0.001). In post-hoc analyses via post oxygenator (Table 2), statistically significant differences were found between median GME counts in control vs. instant (6.0 vs. 322.5, respectively; *p* < 0.0001), in control versus delayed (6.0 vs. 177.0, respectively; p < 0.0001), and in instant vs. delayed (322.5 vs. 177.0, respectively; *p* = 0.0275). Further analysis comparing GME counts across the different pressures post oxygenator (Figure 3) found there was a statistically significant difference in median GME count between pressures (*p* < 0.001). Post-hoc analyses showed statistically significant differences between median GME counts in control vs. −20 mmHg (6.0 vs. 193.0, respectively; *p* < 0.0001) as well as between control versus −40 mmHg (6.0 vs. 338.5, respectively; *p* < 0.0001). However, no statistically significant differences were observed between −20 mmHg and −40 mmHg conditions (Table 3).

**Table 1:** Median GME count by test type (*N* = 50).

Type	*n*	Median	[IQR]
Control	10	6.0	[3.0–15.0]
Instant (−20 mmHg)	10	268.5	[189.0–323.0]
Instant (−40 mmHg)	10	390.5	[247.0–410.0]
Delayed (−20 mmHg)	10	173.0	[137.0–196.0]
Delayed (−40 mmHg)	10	205.0	[140.0–417.0]

**Table 2:** Pairwise comparisons of median GME count by initiation type (*N* = 50).

Type	*p*-value*
Control vs. Instant	<0.0001
Control vs. Delayed	<0.0001
Instant vs. Delayed	0.0275

* Dwass, Steel, Critchlow-Fligner (DSCF) test.

**Table 3:** Pairwise comparisons of median GME count by pressure type (*N* = 50).

Type	*p*-value*
Control vs. −20 mmHg	<0.0001
Control vs. −40 mmHg	<0.0001
−20 mmHg vs. −40 mmHg	0.1985

* Dwass, Steel, Critchlow-Fligner (DSCF) test.

**Figure 4:**
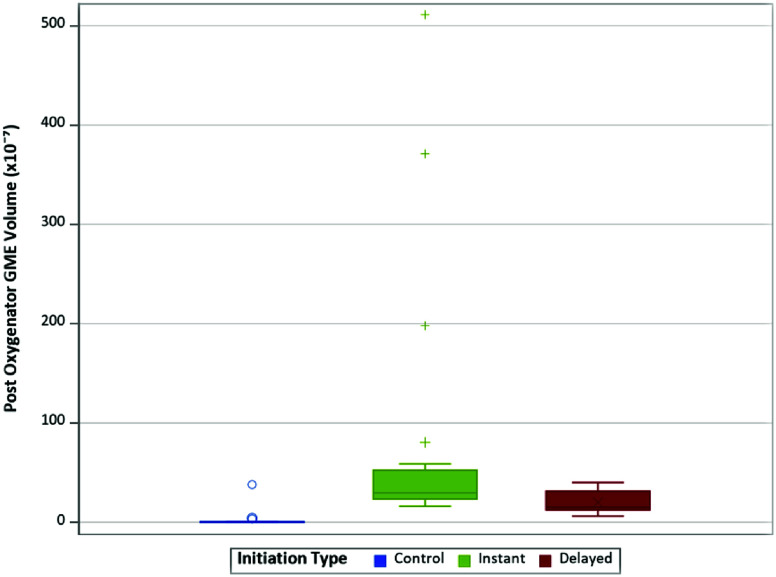
Distribution of post-oxygenator GME volume by initiation type.

**Figure 5:**
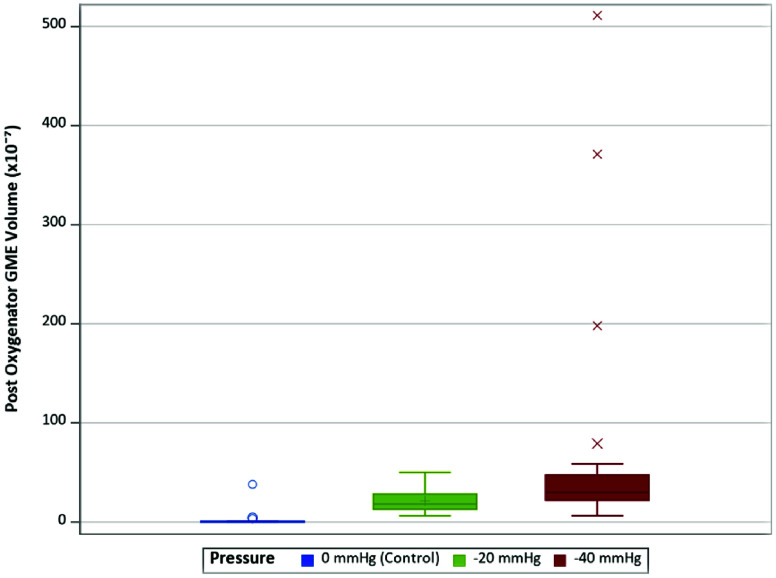
Distribution of post-oxygen GME volume by pressure type.

**Figure 6:**
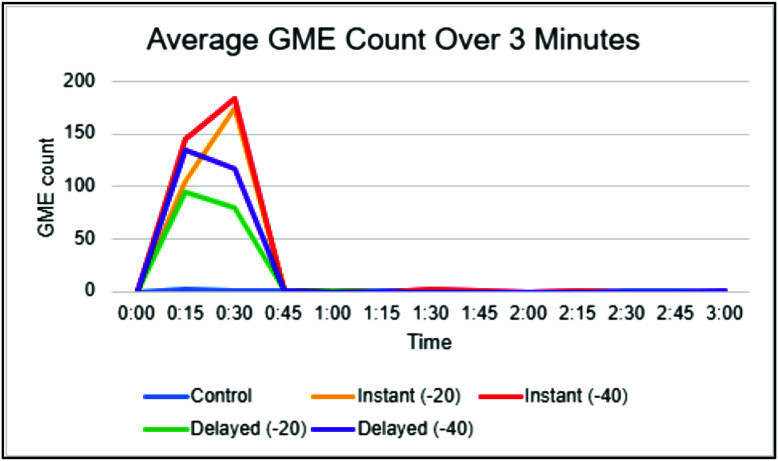
Post-oxygenator average GME count over 3 min.

**Figure 7:**
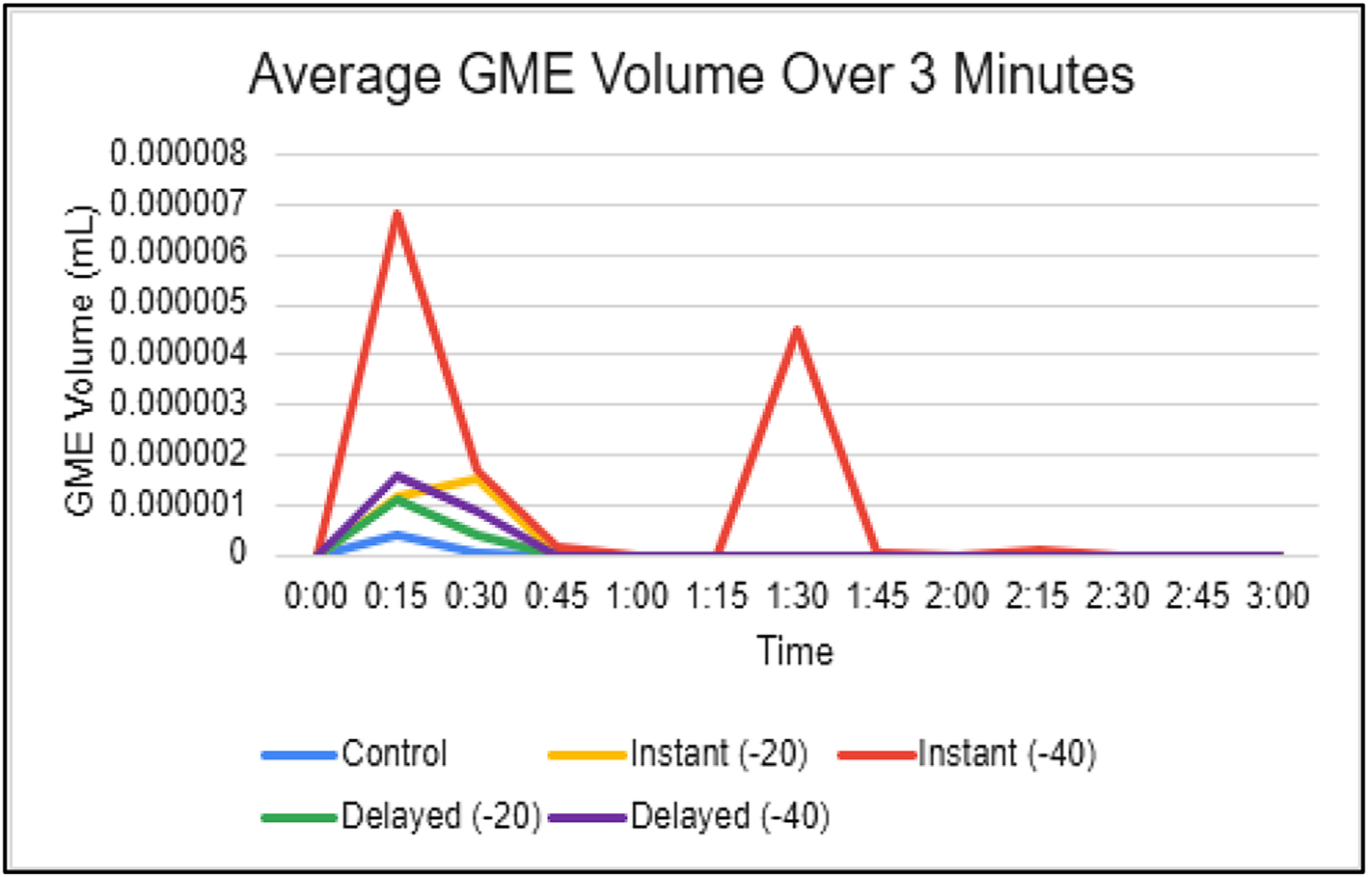
Post-oxygenator average GME volume over 3 min.

**Figure 8:**
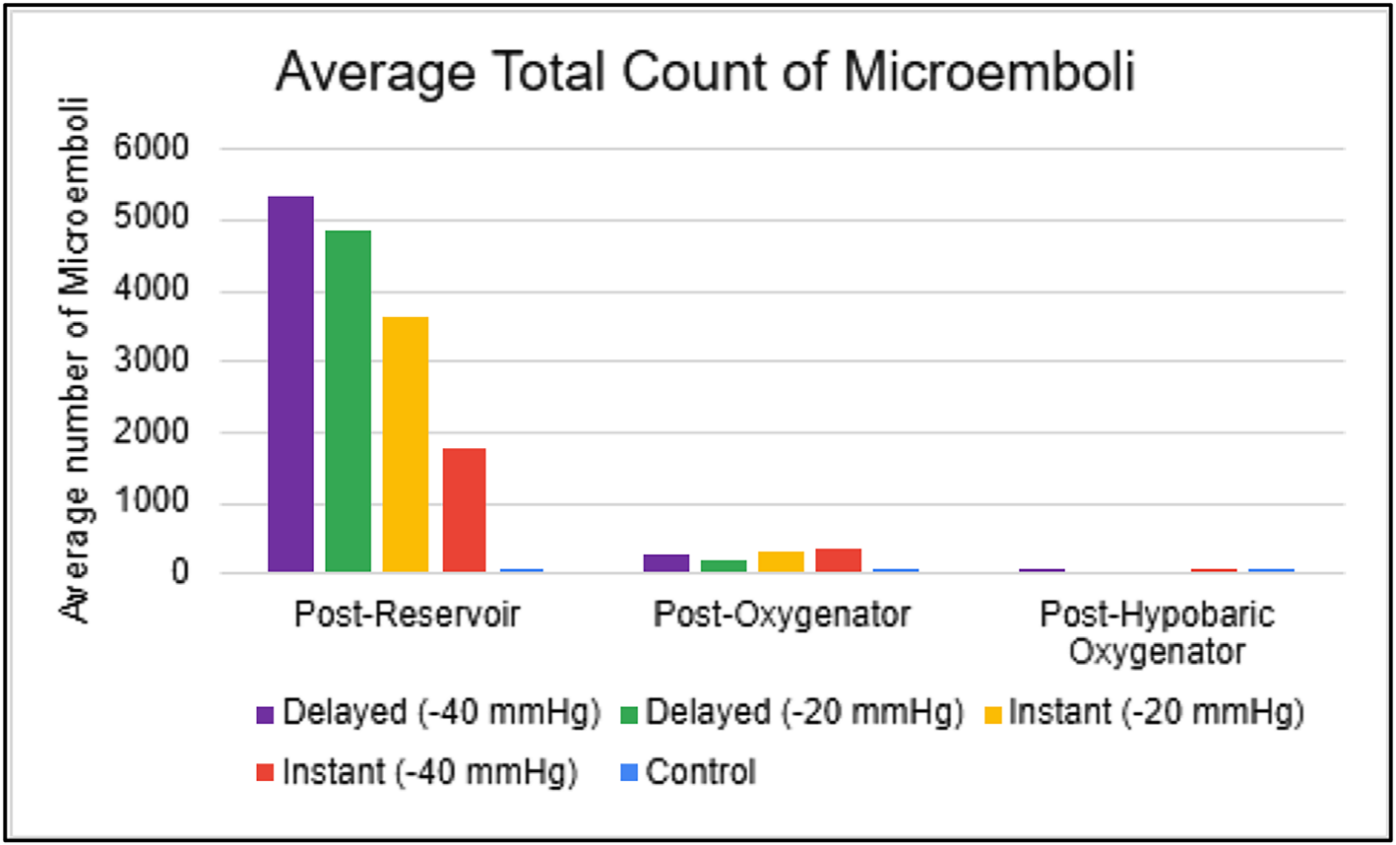
Average GME count pertaining to each group at all EDAC sensors.

**Figure 9:**
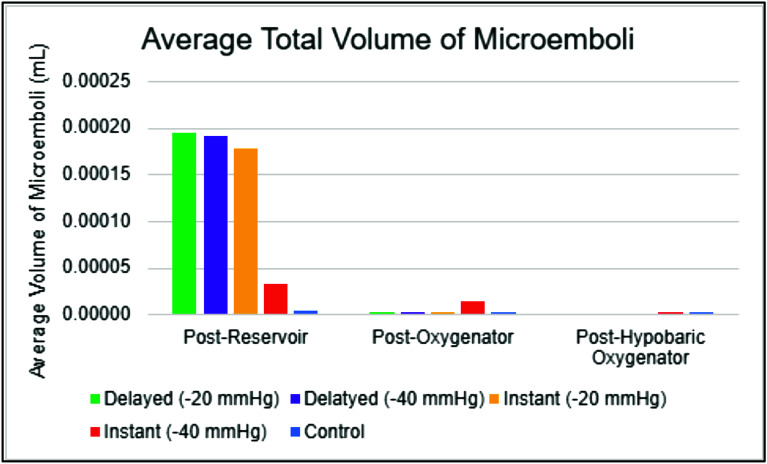
Average GME volume pertaining to each group at all EDAC sensors.

The distribution of post-oxygenator GME volume varied across different experimental conditions (Table 4). The median volume of 0.13 × 10^−7^ for control, whereas the I(−40) condition had a median volume of 43.00 × 10^−7^. Consistent with findings in GME counts, the highest GME volumes were observed at pressures of −40 mmHg within the instant and delayed initiation groups. When assessing post-oxygenator GME volume based on initiation types (Figure 4), there was a statistically significant difference in median volume between types (*p* < 0.001). Post-hoc analyses found statistically significant differences between median GME volume in control versus instant (0.13 × 10^−7^ vs. 29.75 × 10^−7^, respectively; *p* = 0.0004), in control versus delayed (0.13 × 10^−7^ vs. 15.25 × 10^−7^, respectively; *p* = 0.0009), and in instant versus delayed (29.75 × 10^−7^ vs. 15.25 × 10^−7^, respectively; *p* = 0.0037) (Table 5). Additionally, comparing GME volume across the different pressures post oxygenator (Figure 5) found there was a statistically significant difference in median GME volume between pressures (*p* < 0.001). Post-hoc analyses reported statistically significant differences between median GME volume in control versus −20 mmHg (0.13 × 10^−7^ vs. 18.15 × 10^−7^, respectively; *p* = 0.0009), and control versus −40 mmHg (0.13 × 10^−7^ vs. 30.00 × 10^−7^, respectively; *p* = 0.0004). However, no statistically significant difference was observed between −20 mmHg and −40 mmHg conditions (18.15 × 10^−7^ vs. 30.00 × 10^−7^, respectively; *p* = 0.0343) (Table 6). Supporting figures related to data collection can be found in the following figures: GME Postoxygenator average GME count over 3 minutes (Figure 6), Post-oxygenator average GME volume over 3 minutes (Figure 7), Average GME count pertaining to each group at all EDAC sensors (Figure 8), Average GME volume pertaining to each group at all EDAC sensors (Figure 9).

**Table 4:** Median GME volume (×10^−7^) by test type (*N* = 50).

Type	*n*	Median	[IQR]
Control	10	0.13	[0.01–0.72]
Instant (−20 mmHg)	10	24.60	[18.10–30.60]
Instant (−40 mmHg)	10	43.00	[28.70–198.00]
Delayed (−20 mmHg)	10	12.85	[11.00–18.20]
Delayed (−40 mmHg)	10	23.80	[14.30–37.90]

**Table 5:** Pairwise comparisons of median GME volume by initiation type (*N* = 50).

Type	*p*-value*
Control vs. Instant	0.0004
Control vs. Delayed	0.0009
Instant vs. Delayed	0.0037

* Dwass, Steel, Critchlow-Fligner (DSCF) test.

**Table 6:** Pairwise comparisons of median GME volume by pressure type (*N* = 50).

Type	*p*-value*
Control vs. −20 mmHg	0.0009
Control vs. −40 mmHg	0.0004
−20 mmHg vs. −40 mmHg	0.0343

* Dwass, Steel, Critchlow-Fligner (DSCF) test.

## Discussion

These trials found that using a primed venous line resulted in significantly less GME production than that of the experimental groups. Additionally, initiating CPB instantly, rather than waiting for patient volume to reach the reservoir, also creates significantly more GME. Using higher VAVD pressures when initiating CPB with a dry venous line creates more GME within the circuit than at lower vacuum pressures; however, this was not statistically significant. These findings suggest that using a dry venous line technique will create more GME in the circuit (or transmitting to the patient, in practice) than using a traditional primed line. However, GME embolic load may be reduced under the conditions of lower vacuum pressure and slower initiation of CPB, allowing blood from the patient to partially fill the reservoir first. Correlation testing indicates that vacuum pressure contributed more to GME variation between groups than the initiation technique, with an adjusted *R*^2^ of 0.473 for VAVD pressure versus 0.138 for the initiation technique. Together, this represents a 61% correlation between the variables manipulated in this study and GME variation. It is reasonable to conclude that ambient vibrations, imperfect trial standardization, and other confounders account for the other 39% variability in GME production.

For each trial, GMEs were recorded over 3-minute intervals. Notably, nearly all GME detection happened within the first 45 s, and peak detection occurred around 15 s. Interestingly, GME counts peaked later, at around 30 s, for the instant initiation groups. The reason for this delay is not clear. However, one explanation is that it may be due to a delay in GME transmission as the reservoir is still filling during CPB initiation. This differs from the delayed initiation group, which allows more filling (and GME production) before initiation.

Over this interval, an anomaly was observed in GME size for the instant −40 mmHg group. There was a sudden increase in GME size at 1:30 s into the trials for that group. Although this data is an average of 10 trials, no single trial contained a dramatic outlier. An explanation for this is not yet known. Perhaps the increase can be attributed to the air-fluid interaction specific to that group, causing larger GME to be sequestered for longer. Some degree of elevation occurred in multiple trials, which could indicate that there is secondary air leaving the oxygenator in a delayed manner, which would suggest the above theory to be more plausible.

Further, data gathered from the other two sensor locations, post-reservoir, and post-hypobaric oxygenator, provided insight as to the air handling capabilities of the oxygenator and integrated arterial filter. A dramatic reduction in GME size and count between the post-reservoir and post-oxygenator sensors was observed. This affirms the findings by Hudacko and colleagues, who observed significantly less GME post-arterial filter, indicating a high capacity of the filters’ air handling capabilities.

The post-hypobaric oxygenator sensor was used to confirm that no significant air remained in the circuit before beginning each trial. Additionally, it simulated the dissipation of microemboli within the patient shortly after initiating CPB.

### Limitations

Emphasis was placed on standardization between trials, and attempts were made to replicate real clinical conditions using the resources available. However, certain elements of the study differ from the clinical setting. Specifically, 0.9% normal saline was the selected solution for all trials. Although this solution has some osmolar resemblance to human plasma, it does not account for changes in blood viscosity in relation to patient hematocrit. As a non-Newtonian fluid, whole blood maintains a higher viscosity than plasma due to blood cells, platelets, and other clotting factors, but can vary as concentrations change. The higher a patient’s hematocrit, the more viscous their blood becomes. Theoretically, the use of a less viscous fluid allowed for more microemboli formation in the circuit due to air-fluid dynamics.

The use of only one type of extracorporeal circuit, using a centrifugal head versus a roller head arterial pump, and an oxygenator-arterial filter limits the ability to generalize these findings to any given circuit and its components. More research must be conducted to determine if differences exist between extracorporeal devices. Additionally, a built-in arterial filter may have different air-handling capabilities than external arterial line filters, which are still used in some adult clinical practices. Using an arterial roller head as opposed to a centrifugal head may also have affected GME creation. Some movements of the proximal arterial line caused by the roller head motion likely caused more artifact detection than if a centrifugal head were used. Further research on this subject should stipulate the use of whole blood and incorporate a variety of modern extracorporeal circuit equipment.

## Conclusions

The intended use for a dry venous line is to limit the amount of hemodilution that a patient experiences when going on cardiopulmonary bypass. While there is a reservoir present, which theoretically provides protection against air traveling to the patient, the use of a dry venous line combined with vacuum-assisted venous drainage causes an increase in air emboli traveling to the patient. It is important to note that the amount of air observed in this study was very small, but there is no clear answer to the question of “how much air is too much air?”. With that in mind, clinicians should limit patient exposure to GME.

According to published recommendations, there are multiple safe and effective alternatives to limit hemodilution. If a center chooses to practice using a dry venous line, our data suggests that the most effective way to limit GME transmission is to initiate bypass with a reduced vacuum level and a “delayed” initiation approach as detailed above. Further research should be conducted to understand the impact of equipment, disposables, and fluid types.

## Data Availability

The authors confirm that the data supporting the findings of this study are available within the article and its supplementary material. Raw data that support the findings of this study are available from the corresponding author, upon reasonable request.
